# Heterogeneity of Axenfeld–Rieger Syndrome: Molecular and Clinical Findings in Chinese Patients

**DOI:** 10.3389/fgene.2021.732170

**Published:** 2021-10-20

**Authors:** Youjia Zhang, Xueli Chen, Li Wang, Xinghuai Sun, Yuhong Chen

**Affiliations:** ^1^ Department of Ophthalmology and Visual Science, Eye and ENT Hospital, Shanghai Medical College, Fudan University, Shanghai, China; ^2^ NHC Key Laboratory of Myopia, Chinese Academy of Medical Sciences, and Shanghai Key Laboratory of Visual Impairment and Restoration, Fudan University, Shanghai, China; ^3^ State Key Laboratory of Medical Neurobiology and MOE Frontiers Center for Brain Science, Institutes of Brain Science, Fudan University, Shanghai, China

**Keywords:** glaucoma, next generation sequence, genotype-phenotype correlation, *FOXC1* gene, *PITX2* gene, Axenfeld Rieger syndrome

## Abstract

Axenfeld–Rieger Syndrome (ARS) is a rare disease with a wide spectrum of ocular and systemic manifestations. The genetic spectrum of Chinese patients with ARS and genotype-phenotype correlations have yet to be described. To explore the molecular and clinical features in Chinese patients, fifty-five patients clinically diagnosed with ARS from independent families were recruited. Complete ophthalmic examinations and next generation sequencing of anterior segment dysgenesis associated genes were performed in all patients, and segregation in available relatives was verified using Sanger sequencing. 18 *FOXC1* variants, 13 *PITX2* variants, and two gross deletions spanning *FOXC1* were detected in 35 out of 55 (63.6%) patients. 12 *FOXC1* variants, 9 *PITX2* variants, and two gross deletions were novel. There was a wide range of variability and severity in ocular and systemic manifestations displayed in our patients. Patients with *FOXC1* variants were diagnosed at a younger age and had a lower prevalence of systemic manifestations than patients harboring *PITX2* variants and those without variants. To our best knowledge, this is the largest study of Chinese patients with ARS to date. Our findings expand the genetic spectrum of ARS and reveal genotype-phenotype correlations in Chinese patients with ARS. Genetic and clinical heterogeneity were present in our patients. Awareness of the extensive characterization may aid in the clinical management and genetic counseling of patients with this rare disease.

## Introduction

Axenfeld–Rieger Syndrome (ARS; OMIM: 180500, 601499, 602482) is an autosomal dominant developmental disorder that has both clinical and genetic heterogeneity, and primarily affects the anterior segment structure of the eye (Strungaru et al., 2007; Reis et al., 2012). The prevalence of ARS has been estimated to be one in 200,000 individuals (Lewis et al., 2017). ARS comprises a subgroup of anterior segment dysgenesis (ASD) and refers to a constellation of ocular abnormalities, including posterior embryotoxon, iris hypoplasia, corectopia, polycoria, and iridocorneal adhesions (Strungaru et al., 2007; Souzeau et al., 2017). Because of its anomalous anterior segment features, glaucoma is the most serious consequence of ARS, which can lead to irreversible vision loss or even complete blindness (Lewis et al., 2017; Souzeau et al., 2017). Approximately 50% of patients with ARS will develop glaucoma, and treating these patients is difficult (Strungaru et al., 2007). Aside from ocular anomalies, systemic problems also often exist, which typically include dental anomalies, craniofacial abnormalities, and redundant periumbilical skin (Tumer and Bach-Holm 2009; Seifi and Walter 2018). Hearing loss, heart defects, developmental delay, and other variable manifestations have also been reported in patients with ARS (Reis et al., 2012; Seifi and Walter 2018).

*FOXC1* and *PITX2* are two major causative genes of ARS. They are estimated to explain disease pathogenesis in approximately 40–63% of patients with ARS (D’Haene et al., 2011; Reis et al., 2012; Wang et al., 2018; Souzeau et al., 2017). In addition, candidate loci at 13q14 and 16q24 have also been found to be associated with ARS, but no underlying genes have been detected (Chang et al., 2012). *FOXC1* is a member of the large forkhead box (FOX) transcription factor family, whereas *PITX2* is a member of bicoid-like homeodomain transcription factor family. *FOXC1* and *PITX2* are both transcription factors that are coexpressed in the periocular mesenchyme and play key roles in the regulation of embryonic development (Berry et al., 2006).

To date, only limited studies with small sample sizes have been reported in Chinese individuals with ARS. The genotype and phenotype spectrums of Chinese patients with ARS are yet to be described. In this study, we performed a clinical and genetic investigation of 55 Chinese patients with ARS from independent families. We described their clinical ophthalmologic features, presented novel variants in *FOXC1* and *PITX2* genes, and established the genetic and clinical heterogeneity in the present Chinese cohort of patients with ARS.

## Materials and Methods

### Patients

We consecutively recruited 55 patients from independent families that were diagnosed with ARS at the Ophthalmology Department of the Eye and ENT Hospital of Fudan University between December 2004 and June 2020. Both sporadic and familial patients were included. This study was approved by the Institutional Review Board of the Eye and ENT Hospital of Fudan University, and written informed consent was obtained from all patients or their legal guardians. ARS was diagnosed as a group of distinctive ocular features, including malformations of the anterior chamber angle, posterior embryotoxon, iridocorneal adhesions traversing the anterior chamber, corectopia, polycoria, and iris hypoplasia (Alward 2000; Lines et al., 2002). Glaucoma was diagnosed as the presence of at least two of the following criteria: high intraocular pressure (≥ 22 mmHg), glaucomatous optic disc damage, or glaucomatous visual fields defects (Strungaru et al., 2007).

### Clinical Investigation

The family histories and detailed medical histories of the patients were recorded, including the onset and diagnosis ages of ARS and glaucoma, ocular and systemic manifestations, and treatments and their effects. All patients underwent a complete ophthalmologic examination, including visual acuity examination, slit-lamp biomicroscopy, ophthalmoscopy, color fundus photography, gonioscopy, B-mode ultrasonography, A-mode ultrasonography, intraocular pressure (IOP) measurement (Goldmann Applanation Tonometer or Tono-PEN; Reichert, Depew, NY, United States), and ultrasound biomicroscopy (UBM, MD-300L; MEDA Co., Tianjin, China). Perimetry was performed in cooperative children and adults using the Octopus 101 (Haag-Streit, Inc., Köniz, Switzerland) or Humphrey Visual Field Analyzer 750 (Zeiss Humphrey Systems, Dublin, CA, United States).

### Genetic Analysis

Whole blood samples of all patients and available relatives were collected for genomic DNA extraction by Gentra PureGene blood kits (Qiagen, Valencia, CA, United States) according to the manufacturer’s instructions. Genetic testing was performed in 55 patients using next generation sequencing between October 2016 and June 2020. A panel of 289 genes associated with ASD disorders was sequenced by the Illumina Miseq platform (Illumina, San Diego, CA, United States) with the 2 × 300 bp paired-end read module. The average depth was 100x, and 90% of the target region was covered above 40x. Low quality bases (< Q20) were removed using SolexaQA (Cox et al., 2010).

Whole genome sequencing was performed in two patients. In brief, a total of 0.2 μg DNA per sample was fragmented using sonication to a size of 350 bp. The DNA fragments then underwent end-repairing and A-tailing, and ligation was performed with the full-length adapters for Illumina sequencing, followed by polymerase chain reaction amplification and purification. After library quality assessment, clustering of the index-coded samples was performed on the cBot Cluster Generation System using the Illumina PE Cluster Kit (Illumina), and DNA libraries were sequenced on the Illumina platform and 150 bp paired-end reads were generated.

Reads were aligned to the hg19 human reference genome using the Burrows–Wheeler Aligner (BWA; ver. 0.7.11) (Li and Durbin 2009). The detected variants were annotated using ANNOVAR (Wang et al., 2010) and the following databases: the Human Gene Mutation Database (http://www.hgmd.cf.ac.uk/ac/index.php), Clinvar (https://www.ncbi.nlm.nih.gov/clinvar/), and 1,000 Genomes Project (https://www.internationalgenome.org/). The copy number variants were analyzed by calculating the sequencing depth of each region covered by probes. The ExomeDepth Package (Plagnol et al., 2012) was also used to find potential copy number variants. The reference sequences of NM_001453.2 (*FOXC1*) and NM_153427.2 (*PITX2*) were used for mutation nomenclature. Novel variants were classified into five categories according to the American College of Medical Genetics and Genomics (ACMG) guidelines: pathogenic, likely pathogenic, variant of uncertain significance (VUS), likely benign, and benign (Richards et al., 2015). Conservation of the novel variant sites was evaluated using Clustal Omega (Sievers et al., 2011). Polymorphism Phenotyping 2 (PolyPhen2) (Adzhubei et al., 2010), Sorting Intolerant from Tolerant (SIFT) (Kumar et al., 2009), and Provean (Choi and Chan 2015) were applied for the assessment of the pathogenicity of detected missense variants. All detected variants were confirmed using Sanger sequencing, and the segregation on available family members was also verified using Sanger sequencing.

### Statistical Analysis

Statistical analyses were performed using SPSS version 20.0 (IBM-SPSS, Chicago, IL, United States). Age is presented as the median (range). Patients were classified into three groups according to their genotypes. Differences in age with non-normal distributions were assessed using the Kruskal-Wallis test with Dunn’s post-hoc test. Differences between categorical variables among the three groups were assessed using chi-square test or Fisher’s exact test with Bonferroni correction for post-hoc multiple comparisons. The statistical significance was set at *p*-value of 0.05.

## Results

### Patients

Fifty-five Chinese patients with ARS from independent families were enrolled in our study. These included 22 females (40.0%) and 33 males (60.0%). The median age at diagnosis was 14.0 (0.1–64.0) years. 25 patients (45.5%) had family histories of ARS or glaucoma. 53 patients (96.4%) were diagnosed with glaucoma and 36 patients (65.5%) had systemic features.

### Genetic Analysis

**FIGURE 1:**
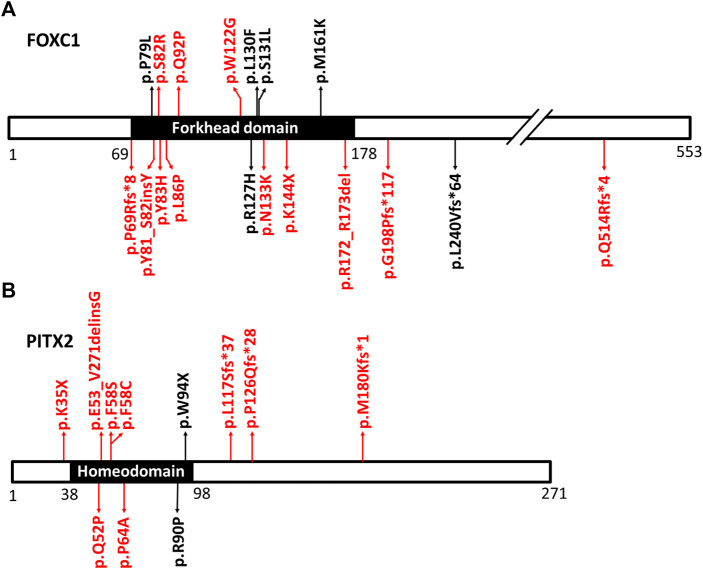
The positions of the detected variants in FOXC1 and PITX2. **(A)** is the diagram of FOXC1 protein and its variants. **(B)** is the diagram of PITX2 protein and its variants. The main function domains (forkhead domain in FOXC1 protein and homeodomain in PITX2 protein) are shown in black. The positions of the variants in FOXC1 and PITX2 are indicated by arrows. The novel variants are shown in red. Gross deletions spanning FOXC1 and splicing variants in PITX2 were not shown in Figure 1.

**FIGURE 2:**
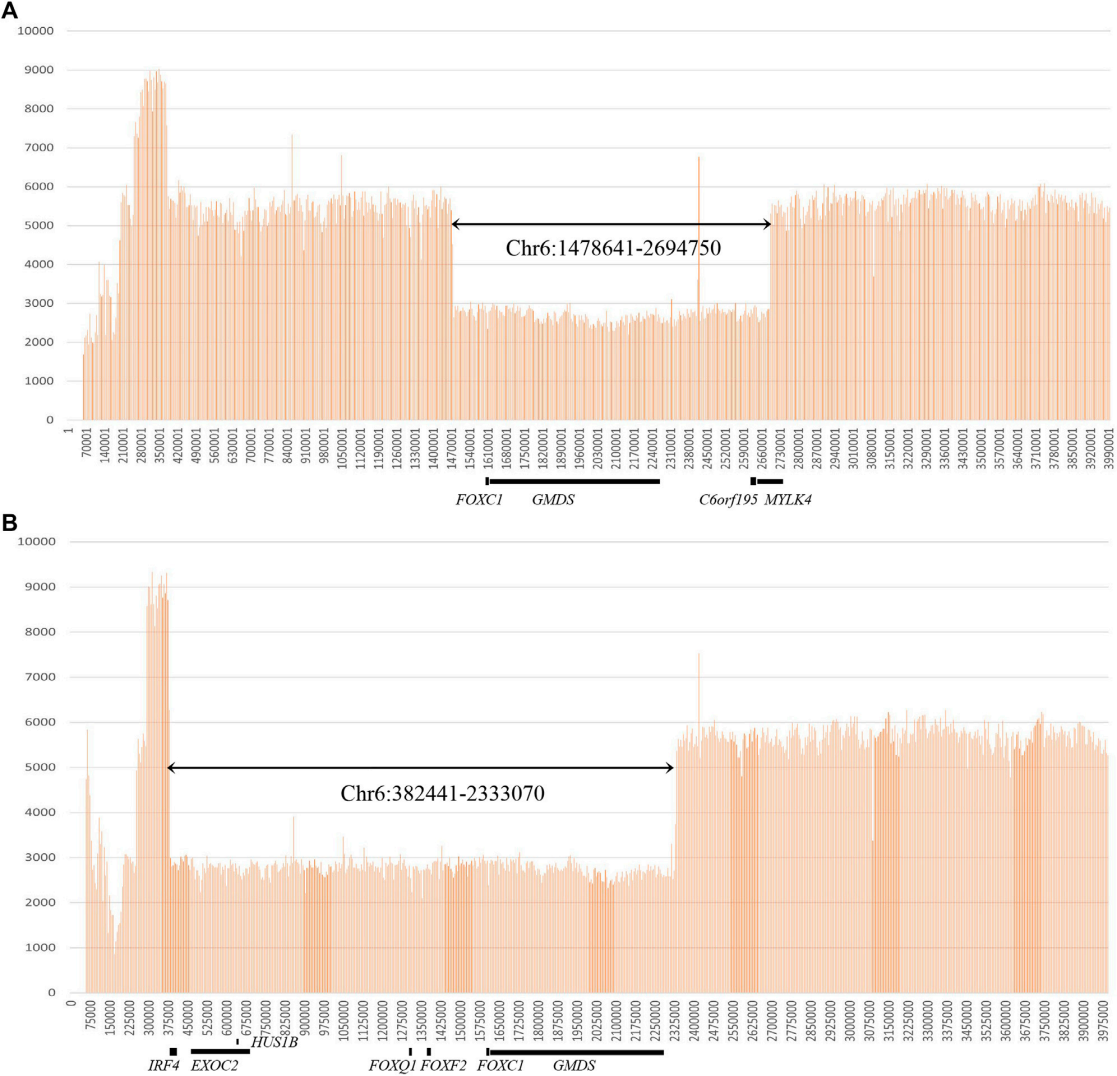
Gross deletions identified by whole-genome sequencing. **(A)** Genome sequencing read depth at the gross deletion (chr6:1478641-2694750) for patient No. 20. **(B)** Genome sequencing read depth at the gross deletion (chr6: 382441-2333070) for patient No. 21.

In total, 33 variants were identified in 35 patients, including 18 variants in *FOXC1*, 13 variants in *PITX2* and two gross deletions of 6p25 (Figures 1, 2). Among them, 12 *FOXC1* variants, 9 *PITX2* variants, and two gross deletions were novel. All detected variants were heterozygous. The variant detection rate was 63.6% (35/55), with 19 patients carrying the *FOXC1* variants, 14 patients carrying the *PITX2* variants, and two patients carrying gross deletions of 6p25. No variants were found in the remaining 20 patients. Among the 35 patients in whom variants were detected, six patients carried *de novo* variants, 11 patients carried heterozygous variants inherited from their parents, and data of parents of 18 patients were unavailable.

Eighteen *FOXC1* mutations and two gross deletions of 6p25 spanning *FOXC1* were detected in 21 patients (21/55, 38.2%) (Table 1). Most of the *FOXC1* variants (15/18) were located in the forkhead domain, which is the DNA interaction function domain of FOXC1 (Figure 1). Six of eighteen *FOXC1* variants have been previously described (Nishimura et al., 1998; Kawase et al., 2001; Nishimura et al., 2001; Panicker et al., 2002; Cella et al., 2006; Strungaru et al., 2007). Other 12 *FOXC1* variants were novel variants, including p.P69Rfs*8, p.Y81_S82insY, p.S82R, p.Y83H, p.L86P, p.Q92P, p.W122G, p.N133K, p.K144X, p.R172_R173del, p.G198Pfs*117, and p.Q514Rfs*4. Ten of them were classified as pathogenic or likely pathogenic and two of them were classified as VUS according to the ACMG classification. All novel missense mutations were predicted to be highly deleterious to the structure and function of *FOXC1* by PolyPhen2, SIFT, and/or Provean (Table 1), located at the evolutionary conserved positions of *FOXC1* corresponding to multiple sequence alignment across species. Heterozygous deletions spanning whole *FOXC1* gene were detected in two patients (No. 20 and No. 21) who presented with both ocular and systemic disorders with very early onset ages. To further determine the exact range of gross deletions in these two patients, whole-genome sequencing was used. By checking the depth of coverage across the whole genome, a large deletion (Chr 6:1478641-2694750) spanning *FOXC1*, *GMDS*, *C6orf195*, and *MYLK4* was detected in patient No. 20, and a large deletion (Chr 6:382441-2333070) spanning *IRF4, EXOC2, HUS1B, FOXQ1, FOXF2, FOXC1,* and *GMDS* was detected in patient No. 21 (Figure 2).

**TABLE 1:** The genotype and phenotype of ARS patients with *FOXC1* mutations or gross deletions of 6p25.

No./Sex/Diagnosis age	Family history^a^	Ocular manifestation	Systemic manifestation	Nucleotide changes^b^	Amino acid changes	Tape of mutation	SIFT/PolyPhen2/Provean	Segregation	ACMG category	Previous literature
1/M/15y	Yes	PE, iridocorneal adhesions, IH, corectopia, GL	—	**c.205delC**	p.P69Rfs*8	Frameshift	NA	Familial; Father/Mother/Brother+	P	Novel
2/F/22y	No	PE, iridocorneal adhesions, GL, high myopia	CHD	c.236C > T	p.P79 L	Missense	Damaging/PD/Deleterious	Sporadic; Father/Mother-	LP	Ref Nishimura et al. (2001)
3/M/4m	No	PE, iridocorneal adhesions, IH, GL (OD), corneal opacification (OD)	—	**c.240_241insTAT**	**p.Y81_S82insY**	Insertion	NA	Sporadic; Father/Mother+	P	Novel
4/F/14y	Yes	PE, iridocorneal adhesions, IH, corectopia, GL	—	**c.246C > G**	**p.S82R**	Missense	Damaging/PD/Deleterious	Familial; Father + Mother/	LP	Novel
5/M/5y	No	PE, IH, corectopia, polycoria, GL, nystagmus, amblyopia	—	**c.247T > C**	**p.Y83H**	Missense	Damaging/PD/Deleterious	Sporadic; Father- Mother-	LP	Novel
6/M/7m	No	PE, iridocorneal adhesions, corectopia, GL, CLDO	—	**c.257T > C**	**p.L86P**	Missense	Damaging/PD/Deleterious	Sporadic; Father- Mother-	LP	Novel
7/M/12y	No	PE, iridocorneal adhesions, IH, GL, keratoconus, corneal decompensation, cataract	—	**c.275A > C**	**p.Q92P**	Missense	Damaging/PD/Deleterious	Sporadic; Father- Mother-	LP	Novel
8/M/7m	No	PE, iridocorneal adhesions, IH, GL, corneal decompensation	CHD	**c.364T > G**	**p.W122G**	Missense	Damaging/PD/Deleterious	Sporadic; Father + Mother/	VUS	Novel
9/M/1m	Yes	PE, iridocorneal adhesions, IH, ectropion uvea, GL	—	c.380G > A	p.R127H	Missense	Damaging/PD/Deleterious	Familial; Father- Mother+	LP	Ref Kawase et al. (2001)
10/F/3m	Yes	PE, iridocorneal adhesions, GL, corneal decompensation	—	c.380G > A	p.R127H	Missense	Damaging/PD/Deleterious	Familial; Father + Mother-	LP	Ref Kawase et al. (2001)
11/M/1m	No	PE, iridocorneal adhesions, GL, corneal opacification	—	c.388C > T	p.L130F	Missense	Damaging/PD/Deleterious	Sporadic; Father- Mother-	P	Ref Strungaru et al. (2007)
12/M/1m	Yes	Iridocorneal adhesions, ectropion uvea, GL, corneal opacification	CHD	c.392C > T	p.S131L	Missense	Damaging/PD/Deleterious	Familial; Father + Mother/	LP	Ref Nishimura et al. (1998)
13/F/15y	Yes	PE, iridocorneal adhesions, GL	—	**c.399C > G**	**p.N133K**	Missense	Damaging/PD/Deleterious	Familial; Father- Mother/	VUS	Novel
14/F/9y	Yes	PE, iridocorneal adhesions, IH, GL, corneal opacification	—	**c.430A > T**	**p.K144X**	Nonsense	NA	Familial; Father- Mother/	LP	Novel
15/M/15y	Yes	PE, iridocorneal adhesions, IH, corectopia, polycoria, ectropion uvea, GL	—	c.482T > A	p.M161K	Missense	Damaging/PD/Deleterious	Familial; Father/Mother/	VUS	Ref Panicker et al. (2002)
16/F/8m	No	PE, iridocorneal adhesions, GL, corneal opacification	—	**c.513_518del**	**p.R172_R173del**	Deletion	NA	Sporadic; Father + Mother-	P	Novel
17/M/2m	Yes	Iridocorneal adhesions, IH, corectopia, polycoria, GL	—	**c.592_593delinsC**	p.G198Pfs*117	Frameshift	NA	Familial; Father + Mother/	P	Novel
18/F/18y	Yes	PE, iridocorneal adhesions, GL	—	c.718_719del	p.L240Vfs*64	Frameshift	NA	Familial; Father/Mother + Sister+	P	Ref Cella et al. (2006)
19/F/40y	No	PE, iridocorneal adhesions, GL, ectropion uvea	Hearing loss	**c.1540delC**	p.Q514Rfs*4	Frameshift	NA	Sporadic; Father/Mother/	P	Novel
20/F/2m	No	PE, iridocorneal adhesions, GL, corneal opacification	CA	**Gross deletion in 6:1478641–2694750**	NA	Deletion	NA	Sporadic; Father/Mother/	P	Novel
21/M/5m	Yes	PE, iridocorneal adhesions, GL	CHD; GD; hydrocephalus	**Gross deletion in 6:382441–2333070**	NA	Deletion	NA	Familial; Father/Mother/	P	Novel

a Note: Family history refers to first-degree relatives having glaucoma or ARS.

b FOXC1 variants were analyzed according to transcript NM_001453.2. Novel variants are shown in bold.

Abbreviations: ACMG, American College of Medical Genetics and Genomics; CA, Craniofacial abnormalities; CLDO, Congenital nasolacrimal duct obstruction; CHD, Congenital heart disease; F, Female; GD, Growth disorder; GL, Glaucoma; IH, Iris hypoplasia; LP, Likely pathogenic; M, Male; NA, Not applicable; OD, Right eye; P, Pathogenic; PD, Probably damaging; PE, Posterior embryotoxon; VUS, Variant of uncertain significance; + , positive for the variant; − , negative for the variant; / , not available for testing.

We also identified 13 *PITX2* variants in 14 patients (25.5%, 14/55) (Table 2), including five missense mutations, three nonsense mutations, two splice site mutations, and three deletions. Four of these have been previously reported (D’Haene et al., 2011; Phillips 2002; Reis et al., 2012; Semina et al., 1996). Most of the variants were located in the homeodomain, which is the DNA binding domain of PITX2 (Figure 1). Nine of these *PITX2* variants were novel variants, including p.K35X, p.Q52P, p.E53_V271delinsG, p.F58C, p.F58S, p.P64A, p.L117Sfs*37, p.P126Qfs*28, and p.M180Kfs*1. All of them were classified as pathogenic or likely pathogenic according to the ACMG guidelines. Four novel missense mutations were located in highly conserved residues and were predicted to be damaging using Provean, PolyPhen2, and/or SIFT*.*

**TABLE 2:** The genotype and phenotype of ARS patients with *PITX2* mutations.

No./Sex/Diagnosis age	Family history^a^	Ocular manifestation	Systemic manifestation	Nucleotide changes^b^	Amino acid changes	Tape of mutation	SIFT/PolyPhen2/Provean	Segregation	ACMG category	Previous literature
22/F/2y	Yes	Iridocorneal adhesions, severe IH, corectopia, polycoria, GL	DA; CA	**c.103A > T**	**p.K35X**	Nonsense	NA	Familial; Father + Mother-	P	Novel
23/F/12y	No	PE, iridocorneal adhesions, IH, polycoria, myopia	DA; UA	**c.155A > C**	**p.Q52P**	Missense	Damaging/PD/Deleterious	Sporadic; Father- Mother-	LP	Novel
24/M/38y	No	PE, iridocorneal adhesions, IH, irregular pupil, GL, cataract	DA	**c.158_173GGTAGCT**	**p.E53_V271delinsG**	Nonsense	NA	Sporadic; Father/Mother/	P	Novel
25/F/10m	No	Iridocorneal adhesions, severe IH, irregular pupil, GL	DA; CA	**c.173T > G**	**p.F58C**	Missense	Damaging/PD/Deleterious	Sporadic; Father/Mother/	LP	Novel
26/F/49y	No	Iridocorneal adhesions, IH, corectopia, GL, corneal opacification (OD)	DA; CA	**c.173T > C**	**p.F58S**	Missense	Damaging/PD/Deleterious	Sporadic; Father/Mother/	LP	Novel
27/M/17y	Yes	PE, iridocorneal adhesions, IH, corectopia, GL, high myopia, cataract	DA; surgery history for umbilical hernia	**c.190C > G**	**p.P64A**	Missense	Damaging/PD/Deleterious	Familial; Father + Mother/	LP	Novel
28/M/34y	No	PE, IH, corectopia, GL	DA	c.253–11A > G	NA	Splicing	NA	Sporadic; Father/Mother/	VUS	Ref Semina et al. (1996)
29/F/46y	Yes	PE, iridocorneal adhesions, IH, corectopia, GL, cataract	DA	c.253–11A > G	NA	Splicing	NA	Familial; Father/Mother/	VUS	Ref Semina et al. (1996)
30/M/24y	No	Iridocorneal adhesions, severe IH, corectopia, irregular pupil, GL	DA; CA	c.253-1G > A	NA	Splicing	NA	Sporadic; Father- Mother-	P	Ref Reis et al. (2012)
31/F/5y	Yes	PE, iridocorneal adhesions, severe IH, corectopia, irregular pupil, GL, corneal opacification, high myopia	CA	c.269G > C	p.R90P	Missense	Damaging/PD/Deleterious	Familial; Father- Mother+	VUS	Ref Phillips (2002)
32/M/64y	Yes	PE, iridocorneal adhesions, IH, corectopia, GL, high myopia, cataract	DA; UA	c.282G > A	p.W94X	Nonsense	NA	Familial; Father/Mother/	LP	Ref D’Haene et al. (2011)
33/M/12y	No	Iridocorneal adhesions, IH, corectopia, polycoria, GL, high myopia	DA; CA; surgery history for umbilical hernia	**c.348delG**	p.L117Sfs*37	Deletion	NA	Sporadic; Father/Mother/	P	Novel
34/M/24y	Yes	PE, iridocorneal adhesions, IH, polycoria	DA	**c.377delC**	p.P126Qfs*28	Deletion	NA	Familial; Father/Mother/	P	Novel
35/M/34y	No	PE, iridocorneal adhesions, IH, corectopia, polycoria, ectropion uvea, GL, high myopia	DA; kidney stone	**c.539_551del**	p.M180Kfs*1	Deletion	NA	Sporadic; Father/Mother/	P	Novel

a Note: Family history refers to first-degree relatives having glaucoma or ARS.

b PITX2 variants were analyzed according to transcript NM_153427.2. Novel variants are shown in bold.

Abbreviations: ACMG, American College of Medical Genetics and Genomics; CA, Craniofacial abnormalities; DA, Dental abnormalities; F, Female; GL, Glaucoma; IH, Iris hypoplasia; LP, Likely pathogenic; M, Male; NA, Not applicable; OD, Right eye; P, Pathogenic; PD, Probably damaging; PE, Posterior embryotoxon; UA, Umbilical anomalies; VUS, Variant of uncertain significance; +, positive for the variant; − , negative for the variant; / , not available for testing.

### Clinical Manifestations and Genotype-Phenotype Correlations

Except for the two patients carrying gross deletions, other patients were divided into three groups: FOXC1 group (patients carrying *FOXC1* variants, n = 19), PITX2 group (patients carrying *PITX2* variants, n = 14), and negative group (patients without *FOXC1* or *PITX2* variants, n = 20). The median age at diagnosis was 5.0 (0.1–40.0) years in the FOXC1 group, 24.0 (0.8–64.0) years in the PITX2 group, and 15.0 (0.3–46.0) years in the negative group. The diagnosis age of the FOXC1 group was significantly lesser than that of the PITX2 group (*p* = 0.006) and the negative group (*p* = 0.048). However, there was no significant difference in the diagnosis age between PITX2 group and the negative group (*p* > 0.99). The ratio of patients that were diagnosed before the age of 1 year was statistically significantly different among the three groups (*p =* 0.007). It was 47.4% (9/19) in the FOXC1 group, but only 7.1% (1/14) in the PITX2 group and 10.0% (2/20) in the negative group.

**FIGURE 3:**
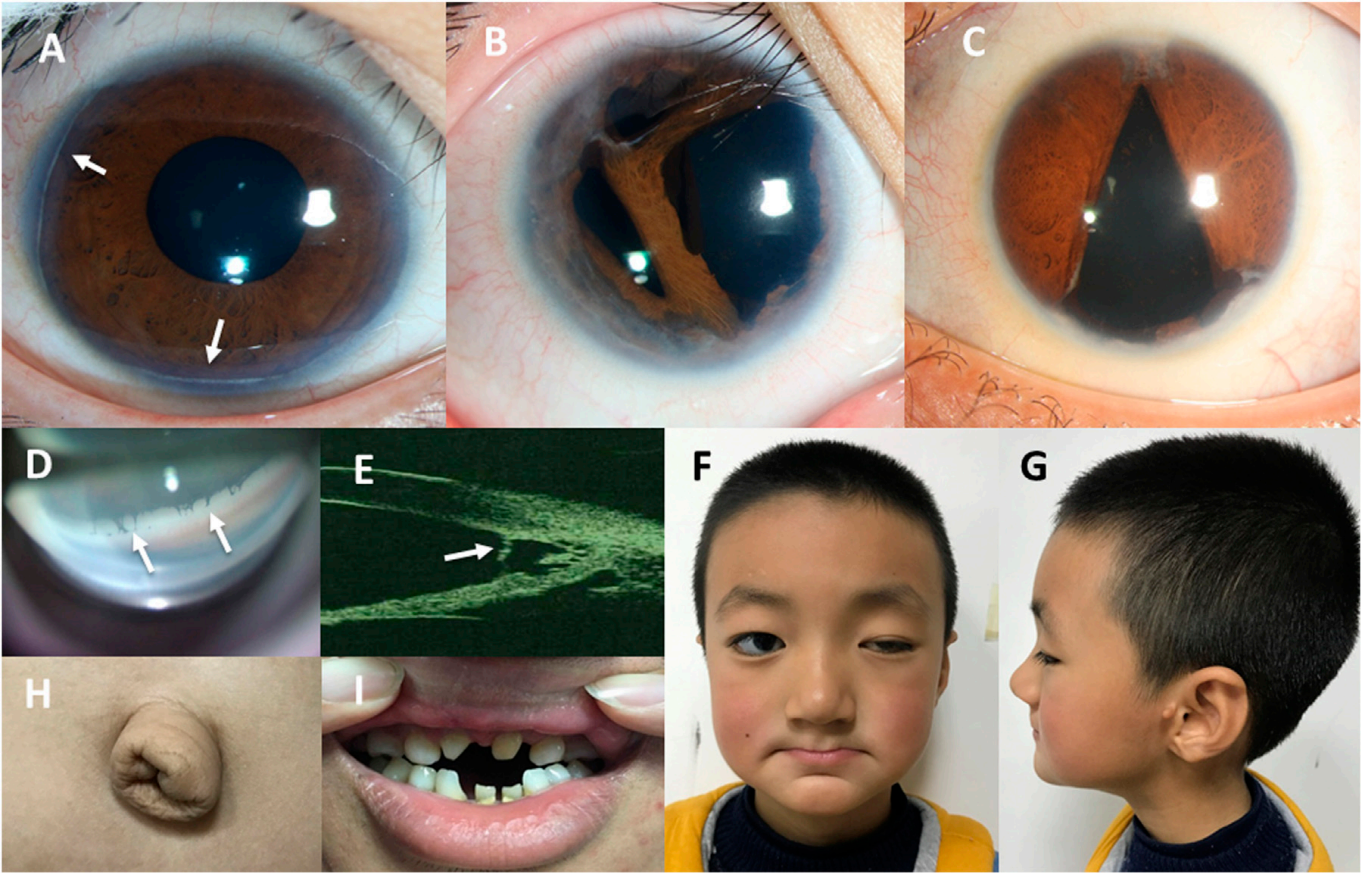
Ocular features and systemic manifestations in our patients with ARS. **(A)** The anterior segment photo of patient No. 13 shows the posterior embryotoxon indicated by white arrowheads. **(B)** The anterior segment photo of patient No. 23 shows iris stromal hypoplasia and polycoria. **(C)** The anterior segment photo of patient No. 45 shows irregular pupil and corectopia. **(D)** The photo of anterior chamber angle under gonioscopy of patient No. 16 shows the iridocorneal adhesions across the anterior chamber angle indicated by white arrowheads. **(E)** The ultrasound biomicroscopy (UBM) image of the anterior chamber angle of patient No. 44 shows the iris strands bridging the iris to the posterior embryotoxon indicated by a white arrowhead. **(F–H)**. The photographs of patient No. 37 shows craniofacial abnormalities (a broad flat nasal root, maxillary hypoplasia, thin upper lip and everted lower lip) **(F,G)** and redundant periumbilical skin **(H)**. **(I)** The photograph of patient No. 41 shows dental anomalies (hypodontia and microdontia).

The patients presented different degrees of anterior chamber and angle anomalies. Iridocorneal adhesion was the most common ocular feature (Tables 1–3), and this presented as iridocorneal tissue adhesions across the anterior chamber angle, observed by gonioscope or UBM (Figure 3). Posterior embryotoxon, which refers to a prominent and centrally displaced Schwalbe’s line (Lines et al., 2002), was another important characteristic (Figure 3). Iris hypoplasia, corectopia, pupillary anomalies, and polycoria were also frequently seen in the patients. Other ocular manifestations included corneal opacification, cataract, corneal decompensation, congenital lacrimal duct obstruction, high myopia, amblyopia, nystagmus, retinal detachment, and exotropia.

**TABLE 3:** The genotype and phenotype of ARS patients without *FOXC1* or *PITX2* mutations.

No./Sex/Diagnosis age	Family history^a^	Ocular manifestation	Systemic manifestation
36/F/3m	No	IH, corectopia, GL	—
37/M/10m	No	Iridocorneal adhesions, severe IH, GL, corneal opacification	DA; CA; UA
38/F/4y	No	PE, iridocorneal adhesions, IH, corectopia, GL, nystagmus, corneal decompensation (OD), amblyopia	DA, hearing loss
39/M/6y	Yes	Severe IH, corectopia, polycoria, GL	DA
40/M/8y	Yes	Iridocorneal adhesions, IH, GL, myopia	DA; UA
41/M/10y	No	IH, corectopia, GL, corneal opacification	DA; CA
42/F/10y	Yes	Iridocorneal adhesions, severe IH, irregular pupil, GL, corneal opacification, cataract	DA
43/F/11y	No	PE, iridocorneal adhesions, GL	—
44/M/14y	No	PE, iridocorneal adhesions, IH, GL, cataract	DA; CA; hearing loss; GD
45/M/14y	No	PE, iridocorneal adhesions, corectopia, irregular pupil, GL, myopia, exotropia	DA
46/M/16y	No	Iridocorneal adhesions, corectopia, GL, cataract, high myopia	—
47/M/18y	No	Iridocorneal adhesions, IH, GL, myopia	DA; CA
48/M/23y	No	Iridocorneal adhesions, IH, irregular pupil, GL, high myopia	DA
49/F/24y	Yes	PE, iridocorneal adhesions, GL	DA
50/M/32y	No	PE, iridocorneal adhesions, IH, GL	DA
51/M/34y	No	PE, iridocorneal adhesions, IH, GL	CA; depressive disorder
52/F/36y	Yes	Iridocorneal adhesions, IH, corectopia, polycoria, GL	DA
53/M/36y	Yes	PE, iridocorneal adhesions, IH, GL	—
54/F/41y	Yes	PE, iridocorneal adhesions, IH, polycoria, GL, corneal opacification	DA, depressive disorder
55/M/46y	Yes	Iridocorneal adhesions, IH, corectopia, polycoria, GL, cataract, high myopia, retinal detachment	DA

a Note: Family history refers to first-degree relatives having glaucoma or ARS.

Abbreviations: CA, Craniofacial abnormalities; DA, Dental abnormalities; F, Female; GD, Growth disorder; GL, Glaucoma; IH, Iris hypoplasia; M, Male; OD, Right eye; OS, Left eye; PE, Posterior embryotoxon; UA, Umbilical anomalies.

As the most frequent consequence of ARS, glaucoma was found in 53 patients (96.4%). Apart from one patient (No. 3) with p.Y81_S82insY in *FOXC1* gene who presented with unilateral glaucoma, all others presented with bilateral glaucoma. Among the patients with glaucoma, 15.1% (8/53) received medication only, 84.9% (45/53) received both medication and surgical treatments, and 60.4% (32/53) underwent at least two surgeries for glaucoma. Three patients carrying *FOXC1* variants (No. 7, No. 8, and No. 10) underwent penetrating keratoplasties because of corneal decompensation.

Furthermore, 36 (65.5%) patients presented with systemic anomalies. The most common systemic manifestations detected in our patients were dental and craniofacial abnormalities. Tooth agenesis presented as hypodontia and microdontia (Figure 3). The craniofacial abnormalities mainly presented as a broad flat nasal root, thin upper lip, and an everted lower lip (Figure 3), which could be seen in almost every ARS patient with facial abnormalities. Other craniofacial features presented as hypertelorism, telecanthus, prominent forehead, and maxillary hypoplasia. Redundant periumbilical skin was also observed in the PITX2 group and negative group. Two patients in the PITX2 group had histories of undergoing surgery for umbilical hernia. Four patients with *FOXC1* variants or gross deletions spanning *FOXC1* had congenital heart diseases. Other systemic manifestations in the patients included hearing loss, growth disorder, depressive disorder, and kidney stone. The prevalence of systemic manifestations was statistically significantly lower in the FOXC1 group (4/19, 21.1%) than in the PITX2 group (14/14, 100%, *p* < 0.001) and in the negative group (16/20, 80.0%, *p* < 0.001); however, there was no significant difference between the PITX2 group and the negative group (*p =* 0.13).

## Discussion

In this study, we investigated 55 Chinese patients with ARS from independent families, and analyzed their genotypes, phenotypes, and genotype-phenotype correlations.

ARS is a rare disease with autosomal dominant inheritance. All of the patients in our study who carried variants are heterozygous. *FOXC1* and *PITX2* were the two major causative genes of ARS, which has genetic heterogeneity. To date, the variant rates of *FOXC1* and *PITX2* in individuals with ARS have been reported in a limited number of studies. In an ASD cohort mainly from Belgium or Netherlands, *FOXC1* and *PITX2* disruptions were recorded in 24% (19/80) and 16% (13/80) of patients, respectively (D’Haene et al., 2011). Whereas, combined *FOXC1* and *PITX2* variants were found to account for 63% of ARS probands (24/38) in a multi-racial study, with 8% (3/38) harboring *FOXC1* variants and 55% (21/38) harboring *PITX2* variants (Reis et al., 2012). In another study of 20 patients with ARS from Southeast China, *PITX2* variants were detected in 55% (11/20) of patients, while no *FOXC1* variants were detected (Wang et al., 2018). The variant rates of *FOXC1* and *PITX2* varied widely among these studies, and this could be due to variations in inclusion criteria and study populations, and limited sample size. In our study, *FOXC1* variants, gross deletions spanning *FOXC1*, and *PITX2* variants in total were detected in 63.6% (35/55) of patients with ARS; *FOXC1* variants were detected in 34.5% (19/55) of probands, gross deletions of 6p25 were detected in 3.6% (2/55) of probands and *PITX2* variants were detected in 25.5% (14/55) of probands. There were no pathogenic variants found in the other 20 patients. The pathogenic genes of these patients still require exploration. By increasing the number of studied patients, we have greatly expanded the genetic spectrum of Chinese patients with ARS.

Eighteen *FOXC1* variants were detected in 19 patients. Most of our detected variants were located in the forkhead domain, which is a conserved 110 amino acid sequence (Figure 1). The forkhead domain is shared by all FOX family proteins and is highly conserved in evolution (Benayoun et al., 2011). The transcription factor encoded by *FOXC1* binds with DNA through the forkhead domain; therefore, the forkhead domain is vital to the localization of FOXC1 to the nucleus, and to the interaction between FOXC1 protein and DNA (Benayoun et al., 2011; Seifi et al., 2017). Forkhead domain variants will impair the translocation, DNA-binding capacity and specificity, and transactivation of FOXC1 (Lehmann et al., 2003; Berry et al., 2006), and ultimately lead to loss of function of FOXC1. FOXC1 is widely expressed in the mesenchyme, and it is important for the regulation of embryogenesis, cell migration, and differentiation (Lehmann et al., 2003; Aldinger et al., 2009; Benayoun et al., 2011; Seifi et al., 2017). Thus, FOXC1 disruptions could lead to abnormal development disorders of the ocular anterior segment and other non-ocular tissues (Lehmann et al., 2003; Chrystal et al., 2021). Deletions are a common form of *FOXC1* variants. To date, 25 small deletions and 35 gross deletions have been detected according to the Human Gene Mutation Database. Similarly, four small deletions and two gross deletions spanning the entire *FOXC1* gene were detected in our patients. Two patients who carried gross deletions had an early age of onset and various multisystemic phenotypes. Patient No. 20 carried a 1.2 Mb deletion, and presented with ocular malformations and craniofacial abnormalities, while patient No. 21 carried a 1.95 Mb deletion, and presented with hydrocephalus, congenital heart disease, and growth disorder, in addition to the ocular malformations. Gross deletions in chromosome 6p25 cause variable clinical features due to the genes involved and the size of the deletions (Fan et al., 2020). Common features include ocular malformations, hydrocephalus, and hearing loss, as well as craniofacial, cardiac, skeletal, and renal malformations (Gould et al., 2004), which were also observed in our patients.

Thirteen *PITX2* variants were found in 14 patients with ARS in our study, including nine novel variants. All missense mutations and two nonsense mutations were located in the 60-amino-acid homeodomain, which is a highly conservative sequence (Figure 1). The homeodomain is responsible for DNA binding and is essential for the activity of the PITX2 protein as a transcription regulator (Priston et al., 2001; Tumer and Bach-Holm 2009). As previously reported, most *PITX2* missense mutations are located in this homeodomain (Tumer and Bach-Holm 2009; Lewis et al., 2017), which could affect all known *PITX2* isoforms and lead to loss of function of the PITX2 protein (Tumer and Bach-Holm 2009; Hendee et al., 2018). Studies using experimental mouse or zebrafish models have demonstrated that *PITX2* plays a key role in embryonic development. Mice with a homozygous null mutation of *Pitx2* were found to have a severe embryonic lethal phenotype with abnormal eye development, defective body-wall closure, abnormal craniofacial development, arrested tooth development, and other numerous abnormalities (Gage et al., 1999; Kitamura et al., 1999; Lin et al., 1999; Lu et al., 1999). Both mice heterozygous for a *Pitx2* null and zebrafish homozygous for a *Pitx2* mutant were found to have ARS-related ocular malformations (Chen and Gage 2016; Hendee et al., 2018). The *PITX2* variants in our patients could disrupt the structure and the transcriptional activity of the PITX2 protein, and ultimately lead to ocular anterior segment and developmental disorders of non-ocular structures.

Our patients displayed a wide range of variability and severity in ocular and systemic manifestations. The spectrum of ARS ocular manifestations mainly included posterior embryotoxon, iris hypoplasia, iridocorneal adhesions, corectopia, and polycoria, consistent with previous studies (Strungaru et al., 2007; Tumer and Bach-Holm 2009; Reis et al., 2012; Souzeau et al., 2017). Due to anterior segment dysgenesis, glaucoma is the most common and serious consequence of ARS. As previously reported, 50–85% of patients with ARS develop glaucoma (Alward 2000; Souzeau et al., 2017; Wang et al., 2018). In our group, 96.4% of patients had glaucoma, and most of them were diagnosed with ARS and glaucoma simultaneously, which was probably because all patients were recruited at glaucoma clinics. The main systemic features in our patients were dental and craniofacial abnormalities. Notably, two patients (No. 27 and No. 33) in the PITX2 group had histories of undergoing surgery for umbilical hernias. It has been reported that redundant periumbilical skin is frequently mistaken for an umbilical hernia (Alward 2000); thus, there was a possibility that these surgeries were unnecessary.

Furthermore, the genotype-phenotype analysis in our study revealed that the *FOXC1* variants were associated with an earlier age of diagnosis and a lower prevalence of systemic features. Patients in the FOXC1 group presented with a significantly earlier age of diagnosis compared to the PITX2 group and the negative group. Furthermore, the proportion of patients aged younger than 1 year was significantly different among the three groups: 47.4% in the FOXC1 group, 7.1% in the PITX2 group, and 10.0% in the negative group (*p* = 0.007). Similarly, Souzeau et al. reported that the age of glaucoma diagnosis was significantly lower in *FOXC1* carriers than in the *PITX2* carriers (Souzeau et al., 2017). Almost half of our patients with *FOXC1* variants presented with ocular manifestation before the age of 1 year, and may present systemic manifestations later in life according to our findings and the literature (Siggs et al., 2019). Thus, long term follow-up and comprehensive physical examination are important in these patients. Interestingly, the prevalence of systemic features in the FOXC1 group was significantly lower than that in the PITX2 group (100%, *p* < 0.001) and in the negative group (80.0%, *p* < 0.001). Furthermore, *FOXC1* was more likely to be associated with congenital heart disease, while *PITX2* was always associated with dental anomalies and/or umbilical anomalies, and this is supported by previous studies (Strungaru et al., 2007; Reis et al., 2012). Our study also illustrates that patients without *FOXC1* or *PITX2* variants had a similar age of diagnosis, and similar prevalence and spectrum of systemic features compared to those harboring *PITX2* variants. This suggests that the undiscovered pathogenic genes of ARS might have a similar function to the *PITX2* gene.

## Conclusion

To our best knowledge, this study enrolled the largest number of Chinese patients with ARS to date. In total, 55 Chinese patients with ARS from independent families were studied both clinically and genetically. We found that 63.6% of patients carried 33 *FOXC1* variants, *PITX2* variants or gross deletions spanning *FOXC1*, out of which 23 were novel. The genotype and phenotype spectrums of Chinese patients with ARS have been greatly expanded by our study. Because of the early age of onset, high risk for glaucoma, and combined systematic disorders, genetic testing is recommended for patients with ARS, in order to make an early and precise diagnosis.

## Data Availability

The data presented in the study are deposited in the LOVD (www.lovd.nl), which is a large Open-Source DNA variation database system. Here is the link: https://databases.lovd.nl/shared/variants/FOXC1?search_var_status=%3D%22Marked%22%7C%3D%22Public%22#object_id=VariantOnTranscript%2CVariantOnGenome&id=FOXC1&search_transcriptid=00008069&search_owned_by_=youjia%20Zhang&page_size=100&page=1; https://databases.lovd.nl/shared/variants/PITX2?search_var_status=%3D%22Marked%22%7C%3D%22Public%22#object_id=VariantOnTranscript%2CVariantOnGenome&id=PITX2&order=VariantOnTranscript%2FDNA%2CASC&search_transcriptid=00024018&search_owned_by_=youjia%20Zhang&page_size=100&page=1.
